# Sow Thistle Chloroplast Genomes: Insights into the Plastome Evolution and Relationship of Two Weedy Species, *Sonchus asper* and *Sonchus oleraceus* (Asteraceae)

**DOI:** 10.3390/genes10110881

**Published:** 2019-11-01

**Authors:** Myong-Suk Cho, Jin Hyeong Kim, Chang-Seok Kim, José A. Mejías, Seung-Chul Kim

**Affiliations:** 1 Department of Biological Sciences, Sungkyunkwan University, Suwon, Gyeonggi-do 16419, Korea; marina0426@gmail.com (M.-S.C.); xopsld@naver.com (J.H.K.); 2 Highland Agriculture Research Institute, National Institute of Agricultural Sciences, Rural Development Administration (RDA), Gangwon-do 25342, Korea; rdacskim@korea.kr; 3 Department of Plant Biology and Ecology, Universidad de Sevilla, 41004 Seville, Spain; jmejias@us.es

**Keywords:** *Sonchus asper* (L.) Hill, *Sonchus oleraceus* L., chloroplast genome evolution, comparative genomic analyses, hybridization, invasive species

## Abstract

Prickly sow thistle, *Sonchus asper* (L.) Hill, and common sow thistle, *Sonchus oleraceus* L., are noxious weeds. Probably originating from the Mediterranean region, they have become widespread species. They share similar morphology and are closely related. However, they differ in their chromosome numbers and the precise relationship between them remains uncertain. Understanding their chloroplast genome structure and evolution is an important initial step toward determining their phylogenetic relationships and analyzing accelerating plant invasion processes on a global scale. We assembled four accessions of chloroplast genomes (two *S. asper* and two *S. oleraceus*) by the next generation sequencing approach and conducted comparative genomic analyses. All the chloroplast genomes were highly conserved. Their sizes ranged from 151,808 to 151,849 bp, containing 130 genes including 87 coding genes, 6 rRNA genes, and 37 tRNA genes. Phylogenetic analysis based on the whole chloroplast genome sequences showed that *S. asper* shares a recent common ancestor with *S. oleraceus* and suggested its likely involvement in a possible amphidiploid origin of *S. oleraceus*. In total, 79 simple sequence repeats and highly variable regions were identified as the potential chloroplast markers to determine genetic variation and colonization patterns of *Sonchus* species.

## 1. Introduction

Prickly (or spiny) sow thistle, *Sonchus asper* (L.) Hill, and common (or annual) sow thistle, *S. oleraceus* L., are two well-known worldwide noxious weeds. The seeds can germinate throughout the year over a broad range of temperatures [1,2]. These species are considered to be particularly troublesome weeds across the grain growing regions because they have allelopathic potential to function as interference competition even in weed communities [3] and are prolific seed producers (up to 25,000 seeds per single plant in a fallow); the seeds possess pappi, which helps wind-mediated dispersal [4]. Moreover, the evolution of herbicide resistance found that populations of these species threatens the efficiency of weed control. For example, several populations of *S. oleraceus* have been reported as resistant to acetolactate synthase (ALS) inhibiting herbicides including chlorsulfuron (Group B) and glyphosate herbicides (Group M) in Australia [5,6]. In addition to being troublesome weeds worldwide, the tender leaves of common sow thistles are consumed as salads and potherbs and the extracts of both sow thistles have been used as medicinal herbs in Brazil, New Zealand, and China [7,8].

Prickly and common sow thistles belong to the subgenus *Sonchus* of the genus *Sonchus* L. (Asteraceae); the genus is a member of the subtribe Hyoseridinae Less. (formerly known as Sonchinae sensu K. Bremer) [9,10]. The current, redefined genus *Sonchus* in its wider circumscription is comprised of ca. 95 species, which are widely distributed globally. They mainly grow in native ranges from most of the Old World (Eurasia and Africa) and mid-Atlantic islands, avoiding the coldest and driest regions, and extend to Australia/New Zealand and the Southeast Pacific islands of the Juan Fernández and Desventuradas [9,10]. Almost half of the species diversity is found in the oceanic insular ranges, which are examples of spectacular adaptive radiation (Macaronesia in the Atlantic and the Juan Fernández and Desventuradas in the Pacific) [11,12,13]. Some of the most common species, both prickly and common sow thistles, have been introduced to America, being particularly widespread in northern temperate regions. The genus seems to have originated in the western Mediterranean region, including areas of western Morocco with a Mediterranean-type climate. The geographical origins of weedy sow thistles have not been formulated with any certainty, but they are also believed to have originated in the Mediterranean region, becoming one of the cosmopolitan species on planet Earth [14]. Both species are widespread and prevalent in Europe, Asia, Africa, and have also been introduced to America and Australia [1,11,15]. They are pioneer species, invade mainly open and disturbed areas, and occur in a wide variety of environments like cultivated fields, gardens, roadsides, pastures, and waste places.

**Figure 1:**
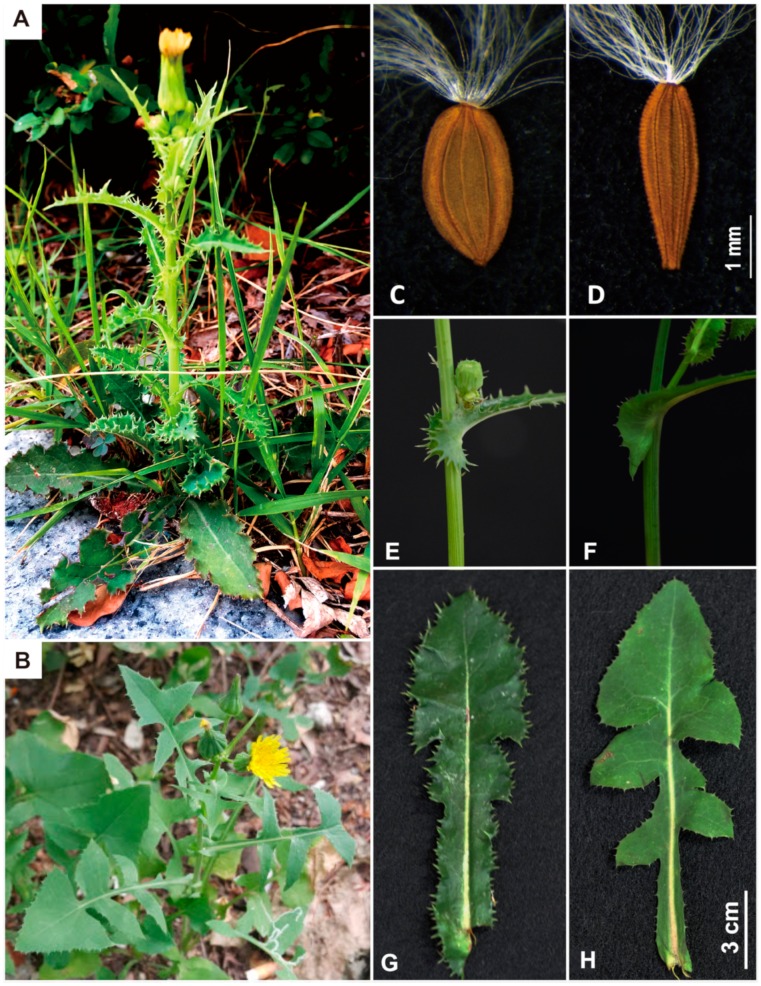
Pictures of *Sonchus asper* (prickly sow thistle) and *Sonchus oleraceus* (common sow thistle). *Sonchus asper* (**A**). plant; (**C**). achene; (**E**,**G**). rounded leaf base auricle and prickly leaf margin. *Sonchus oleraceus* (**B**). plant; (**D**). achene; (**F**,**H**). pointed leaf base auricle and lobed leaf margin. Photo credits: (**C**,**D**) (Jose Mejías); (**A**,**B**,**E**–**H**) (Jin Hyeong Kim and Myong-Suk Cho).

Although some diagnostic features are relevant for species identification, *S. asper* and *S. oleraceus* can sometimes be confused morphologically. Both species are erect herbs, annual or biennial weeds, and have yellow florets and hollow and smooth stems. They can be either low-growing rosettes or upright in their growth form and overwinter as achenes, but fall-germinated plants may overwinter as rosettes in milder climates. Despite their similar morphology and phenology, *S. asper* and *S. oleraceus* can be distinguished primarily based on the characteristics of their leaves and fruits (Figure 1). The leaves of *S. asper* are deep, shiny, and green, rarely divided, thick, and have very prickly leaf margins, while those of *S. oleraceus* are flatter, pinnatifid, and weakly spinous. In addition, the leaf base auricles of *S. asper* are often recurved or curled and rounded, while those of *S. oleraceus* are deltate to lanceolate, almost straight, and acute. However, the characteristic of leaf base and degree of prickly dentate leaf margin can be variable in both species, often confusing species identification based on these traits only. The most consistent reproductive diagnostic features are in their fruits: the cypselae of *S. oleraceus* are slightly compressed, with rounded margins, (2)3(4) ribs per side, two of them furrowed lengthwise, and transversely rugulose or tuberculate across and between ribs, while those of *S. asper* are strongly compressed, more or less winged, 3 ribs per side, and smooth across and between the ribs [4] (Figure 1). One important karyotype characteristic is their different ploidy levels. *S. asper* has been represented uniformly by diploid plants (somatic chromosome number 2*n* = 18) in Europe, USA, and Canada, while *S. oleraceus* has typically been reported with a chromosome number of 2*n* = 32 in Eurasia, Africa, and North America [11,16,17,18,19,20]. However, descending aneuploid (2*n* = 16; from Eurasia) and tetraploid (2*n* = 36; from North Dakota, USA) chromosome counts also have been rarely reported [16], although the taxonomic identity of the plant material studied remains unclear. Stebbins et al. [21] proposed an amphidiploid origin for *S. oleraceus* (*n* = 16, 2 *n*= 32), which has combined *S. asper* (*n* = 9, 2*n* = 18) and *S. tenerrimus* L. (*n* = 7, 2*n* = 14). Certainly, the morphological characteristics of *S. oleraceus* reflect a remarkable middle ground between the two putative parental species. It is also plausible that the hybrid origin of *S. oleraceus* increased ecological amplitude and evolutionary success via (re-)combining two different genomes. Previous molecular phylogenetic studies [13,22,23,24] provided evidence that *S. asper* and *S. oleraceus* are closely related to each other, but failed reciprocal monophyly due to poor resolution. It is of great interest to determine the origin of amphidiploid *S. oleraceus* based on extensive sampling strategy and rigorous molecular and cytological tools.

Chloroplasts in plant cells serve as metabolic centers and encode many key proteins that are involved in photosynthesis and other metabolic processes, primarily participating in photosynthesis, transcription, and translation [25]. Chloroplast (cp) sequence polymorphisms have been extensively used as useful genetic markers at wide ranges of taxonomic levels in plants. They have provided valuable insights into phylogenetic relationships and the origin and evolution of the crop species [26] or introduced/invasive species [27,28,29,30] that are facilitated by maternal inheritance of cp genomes. However, earlier phylogenetic analyses utilizing the partial cpDNA sequences from several regions often resulted in limited sequence variation owing to highly conservative genome evolution, particularly for closely related and recently radiated groups of species [31]. The advent of high-throughput sequencing technologies of next-generation sequencing (NGS) has helped to reveal considerable genome-wide variations in terms of sequences and structures of entire chloroplast genomes. The benefits of genome-wide data have increased phylogenetic resolution and significantly enhanced our understanding of plant evolution and diversity in the field of chloroplast genetics and genomics [25]. The chloroplast phylogeny based on several coding and noncoding cpDNA regions in previous studies has not provided enough resolution to elucidate the phylogenetic relationship among weedy *Sonchus* species as well as the origin of *S. oleraceus*. In both of the cpDNA and nuclear DNA phylogenies, *S. oleraceus* was more closely related to *S. asper* than to *S. tenerrimus*, even though the precise relationship between them was not clear [13,22,23,24]. Taking cpDNA phylogeny and maternal inheritance into consideration, it could be hypothesized that *S. asper* contributed as the maternal parent in the origin of amphidiploid *S. oleraceus*. To test this hypothesis and to better understand the evolutionary relationships of two weedy sow thistle species, we characterized four accessions of complete chloroplast genomes (two from *S. asper* and two from *S. oleraceus*) and conducted the comparative analyses of their whole chloroplast genomes. All but one accession of *S. oleraceus* was collected from the probable origin of diversity in the Mediterranean region. Considering their global distribution, one accession of *S. oleraceus* was collected from Dok-do Island in Korea and one more previously published accession of *S. oleraceus* from Australia (GenBank accession number MG878405) was also included in the comparative analyses. 

## 2. Materials and Methods 

### 2.1. Material Preparation, DNA Extraction, Genome Sequencing, and Annotation

**Figure 2:**
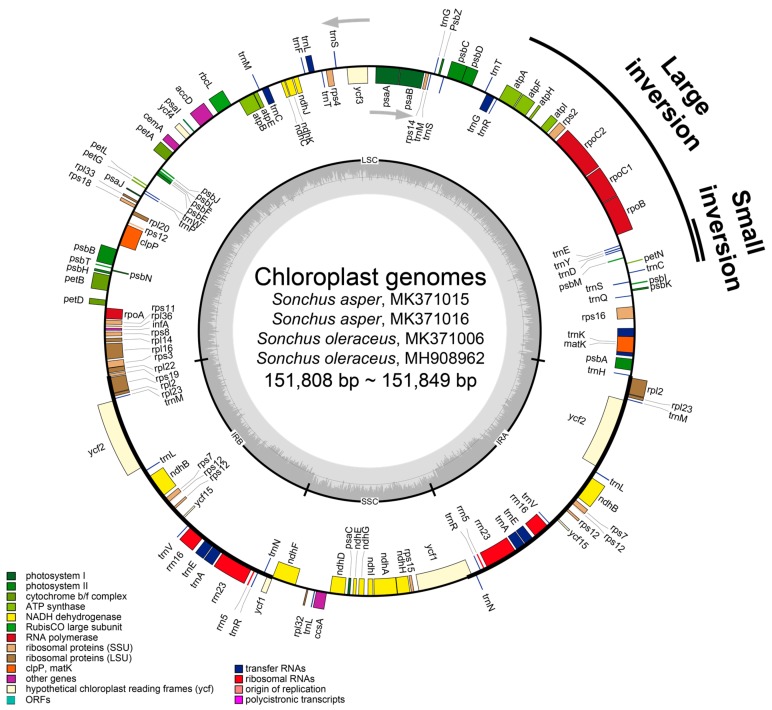
Merged gene map of four weedy *Sonchus* chloroplast genomes of two accessions each of *S. asper* and *S. oleraceus* that were sequenced in this study. The genes inside and outside of the circle are transcribed in the clockwise and counterclockwise directions, respectively. Genes belonging to different functional groups are shown in different colors. The thick lines indicate the extent of the inverted repeats (IR_A_ and IR_B_) that separate the genomes into small single copy (SSC) and large single copy (LSC) regions. Large inversion and smaller inversion nested within the large inversion that is unique in Asteraceae are indicated with black lines outside the gene map.

The silica-gel dried leaves of four prickly and common sow thistles which were sampled from natural habitats in Spain and Korea were used as sources of DNA. Two accessions of *S. asper* were sampled from Seville (VIL-1) and Huelva (MAR-1) in Spain, while *S. oleraceus* was sampled from Huelva (MAR-1) in Spain and Dok-do Island in the East Sea, Korea. Total genomic DNA was isolated using the DNeasy Plant Mini Kit (Qiagen GmbH, Hilden, Germany) following the manufacturer’s protocol. An Illumina paired-end (PE) genomic library was constructed and sequenced using the Illumina HiSeq platform (Illumina, Inc., San Diego, Ca, USA) at Macrogen Corporation (Seoul, Korea). The sequence reads of chloroplast genomes were assembled by the de novo genomic assembler, Velvet 1.2.10 [32] at coverages ranging from 789x to 1354x. Annotation was performed using the Dual Organellar GenoMe Annotator [33], ARAGORN v1.2.36 [34], and RNAmmer 1.2 Server [35]. Using Geneious v8.1.6 (Biomatters Ltd., Auckland, New Zealand), the draft annotation was inspected and corrected manually, performing blast search by comparison with homologous genes in *Lactuca sativa* (DQ383816), *S. oleraceus* (MG878405), *S. canariensis* (NC042381), *S. acaulis* (NC042382), and *S. webbii* (NC042383) from the GenBank database at the National Center for Biotechnology Information (NCBI) as references. The complete plastome sequences were registered in GenBank under the accession numbers MH908962 (*S. oleraceus* from Korea, Collection # Son0-uDS2), MK371006 (*S. oleraceus* from Spain, Population # MAR-1), MK371015 (*S. asper* from Spain, Population # VIL-1), and MK371016 (*S. asper* from Spain, Population # MAR-1). In addition, the raw HiSeq reads were deposited in the Short Read Archive (SRA) at NCBI under Bioproject ID PRJNA0577793 for MH908962 and PRJNA578572 for MK371006, MK371015, and MK371016. OGDRAW [36] was used to draw circular chloroplast genome maps (Figure 2).

### 2.2. Repeat Sequence Analysis

Two types of repeat sequences were identified in the five chloroplast genomes of *S. asper* and *S. oleraceus* including previously reported *S. oleraceus* from Australia (GenBank accession number MG878405). REPuter [37] was used to detect the various types of repetitive sequences of the five *Sonchus* chloroplast genomes. Search parameters were set to: maximum computed repeats = 50, minimum repeat size = 8 bp, and hamming distance = 0. Simple sequence repeats (SSRs) were identified using MISA web (http://pgrc.ipk-gatersleben.de/misa/) with search parameters of 1–15 (unit size-minimum repeats, i.e., mono-nucleotide motifs with 15 minimum numbers of repetition), 2-5, 3-3, 4-3, 5-3, and 6-3 with 0 interruption (maximum difference for two SSRs).

### 2.3. Identification of Highly Divergent Regions

The five weedy *Sonchus* chloroplast genomes were compared to the reference genome of *S. webbii* at the entire chloroplast genomic level using DnaSP [38] and mVISTA [39]. *S. webbii* is a herbaceous perennial unlike other woody *Sonchus* species (*S. acaulis* and *S. canariensis*) in the Canary Islands and is sister to the clade containing five accessions of weedy *Sonchus* species. Overall sequence divergence was estimated for the five weedy *Sonchus* chloroplast genomes that were aligned and compared to the reference genome using the LAGAN alignment mode [40] in mVISTA. Nucleotide diversity (Pi) was calculated using the sliding window analysis (window length = 1000 bp and step size = 200 bp excluding sites with alignment gaps) to detect the most divergent regions (i.e., mutation hotspots) among the five weedy *Sonchus* genomes in DnaSP. The borders of large single copy (LSC), small single copy (SSC), and inverted repeats (IRs) were compared with the results of DnaSP and mVISTA.

### 2.4. Phylogenetic Analysis

To investigate the taxonomic position and phylogenetic relationship of the newly sequenced accessions of *S. asper* and *S. oleraceus*, 26 complete chloroplast sequences representing major lineages of the family Asteraceae were obtained from GenBank. In total, full sequences of 30 chloroplast genomes were aligned using MAFFT v.7 [41]. A maximum likelihood tree was produced based on the relationships of whole chloroplast genomes by IQ-TREE [42] with 1000 replicate bootstrap (BS) analyses. The best fit evolutionary model was chosen as TVM + F + I + G4, which was scored according to the Bayesian information criterion (BIC) scores and weights by using ModelFinder [43] implemented in IQ-TREE.

## 3. Results and Discussion 

### 3.1. Comparative Genomic Analysis of Five Weedy Sonchus Chloroplast Genomes in Content, Order, and Organization

**Figure 3:**
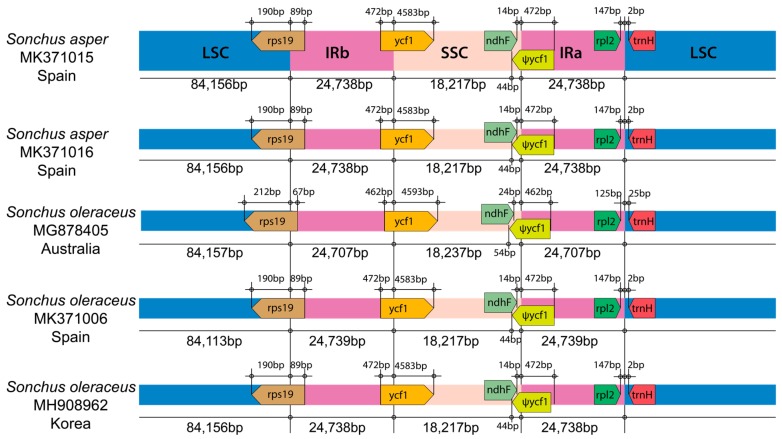
Comparison of the border positions of the large single copy (LSC), small single copy (SSC), and inverted repeat (IR) regions among five weedy *Sonchus* chloroplast genomes representing *S. asper* and *S. oleraceus*. Gene names are indicated in the boxes and their lengths in the corresponding regions are displayed above the boxes. Ψ indicates a pseudogene.

Despite morphological and cytological differences between two weedy *Sonchus* species (*S. asper* and *S. oleraceus*), gene content and arrangement were found to be identical in five chloroplast genomes, displaying 99.98% pairwise similarity in sequences (Table 1). The total length of five cp genomes ranged from 151,808 (*S. oleraceus* from Spain and Australia) to 151,849 (the two samples of *S. asper* from Spain and *S. oleraceus* from Dok-do, Korea) base pairs (bp) and consisted of four typical regions: LSC, SSC, and a pair of inverted repeat regions (IR_A_ and IR_B_). One large inversion with a size of 22.8 kb and a second smaller inversion of 3.3 kb, nested within the large inversion, were found in all five chloroplast genomes (Figure 2). These inversions are unique to Asteraceae and present in most of the family, but are absent in the species of the basal subfamily Barnadesioideae [44,45]. The overall guanine-cytosine (GC) content of each chloroplast genome was 37.6%, with LSC, SSC, and IR regions having 35.8%, 31.4–31.5%, and 43.1% GC contents, respectively. Each of the five cp genomes contained 130 genes, including 80 protein-coding genes (plus seven duplicated in IR), three rRNA genes (all duplicated in IR), and 30 tRNA genes (plus seven duplicated in IR). Eighteen genes contained introns, including seven tRNA genes. Three genes of *clp*P, *rps*12, and *ycf*3 exhibited two introns. The *trn*K tRNA gene harbored the largest intron, which contained the *mat*K gene in between. In total, 17 genes were duplicated in the IR regions, including seven tRNAs, three rRNAs, and seven protein genes. The trans-splicing gene *rps*12, consisting of three exons, was located in the LSC region for exon 1, but exon 2 and exon 3 of the gene were imbedded in the IR regions. Part of *ycf*1 and *rps*19, for which we did not annotate in the four weedy *Sonchus* cp genomes that were sequenced in this study, were duplicated in the IR_A_ region, forming pseudogenes. A pseudo *ycf*1 gene in IR was extended to the SSC region and overlapped with the near *ndh*F gene (Figure 2 and Figure 3; Table 1 and Table 2). The genomic features of five weedy *Sonchus* genomes were nearly identical to congeneric species in the woody *Sonchus* alliance in the Macaronesian Islands (Atlantic Ocean), *S. canariensis* (NC042381), *S. acaulis* (NC042382), and *S. webbii* (NC042383) in gene content and overall GC rate [46]. The cp genomes of other Asteraceae species were also highly conservative in gene order and content with minor variations in the gene prediction of several genes (e.g., *ycf*68 protein coding gene, 4.5 rRNA genes and pseudogenes), even though they represent morphologically and genetically diverse tribes of Asteraceae belonging to Anthemideae, Cardueae, Cichoroieae, Eupatorieae, Heliantheae, Millerieae, and Senecioneae [47,48,49]. 

### 3.2. SSRs and Large Repeat Sequences

**Figure 4:**
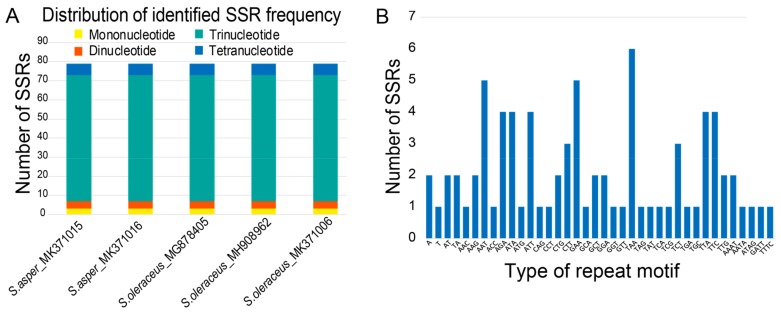
Simple sequence repeat (SSR) number per distribution and repeat type of five accessions of weedy *S. asper* and *S. oleraceus* chloroplast genomes. (**A**) Variation in the numbers of SSRs detected in five chloroplast genomes of weedy *S. asper* and *S. oleraceus*. (**B**) Number of SSR motifs in different repeat motifs of each weedy *Sonchus* chloroplast genome.

Repeat sequences are considered to play an important role in genome recombination and rearrangement [50,51]. Particularly, SSRs, which represent a unique type of tandemly repeated genomic DNA sequence, have high polymorphisms due to large variations in motifs and number of repetitions. Because of the high level of polymorphisms and genome-wide distribution, they have been considered as powerful tools to measure genetic diversity and address population genetic issues at the level of inter- and intra-specific variations, such as gene flow, parentage, and population structure [52]. We found that all the five cp genomes contained the same numbers and distribution patterns of repeated sequences in each cp genome. There were 79 SSRs detected by MISA [53] based on search parameters set for 1–15 (mono-nucleotide motifs with 15 minimum numbers of repetition), 2–5, 3–3, 4–3, 5–3, and 6–3. The majority of the SSRs were tri-nucleotide motifs (66 SSRs, 84%). Remarkably, there were low proportions of other SSR types, i.e., three mono-nucleotide SSRs (4%), four di-nucleotide SSRs (5%), and six tetra-nucleotide SSRs (7%) (Figure 4A). The abundance of tri-nucleotide SSRs was consistent with previous findings with similar parameter settings [54]; however, the frequency of mono-nucleotide SSRs was significantly lower because of the more stringent search parameter used in this study (i.e., minimum repeat of 15) than in previous studies (minimum repeat of 8 or 10) [47,54]. The most abundant repeat motif was “AAT/ATT” followed by “AAG/CTT” in all five genomes (Figure 4B, Table S1). Interestingly, SSRs were distributed most abundantly in the coding regions (57%), followed by intergenic regions (38%), however much lower numbers were distributed in the non-coding introns (5%) in each cp genome. The coding regions with the highest number of SSRs were *ycf* genes as shown in *Cynara cardunculus* (globe artichoke) and other Asteraceae species [47]. Gene *ycf*2 contained 10 SSRs (five duplicated in each IR) and *ycf*1, three in SSC, emphasizing that these highly variable regions can be of specific interest to develop future cpSSR markers for phylogenetic studies of *Sonchus* species. Considering the quadripartite regional occupancy of SSRs, the IR and SSC regions were remarkably lower in overall SSR frequency compared with the LSC region: 19% from the SSC region and 10% from each of the two IR regions versus 61% from the LSC region (Table S2). 

**Figure 5:**
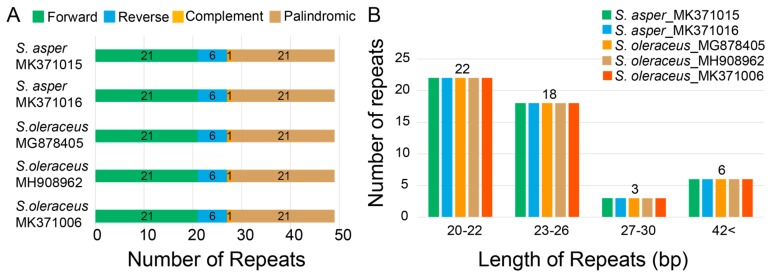
Repeat numbers per repeat type and repeat length of five chloroplast genomes of two *Sonchus* species. (**A**) Variation in the distribution of forward, reverse, complement, and palindromic repeats in each chloroplast genomes of five weedy *Sonchus* genomes. (**B**) Number of different repeat lengths of each weedy *Sonchus* chloroplast genomes.

In the case of the large repeats, we found 49 pairs in each cp genome using the parameters of maximum computed repeats = 50, minimum repeat size = 8 bp, and hamming distance = 0 by REPuter [37]. They contained 21 forward, 6 reverse, 1 complement, and 21 palindromic matches of repeats (Figure 5A). Similar results were reported in previous studies with the majority of repeats in forward (21) and palindromic (13) in other Asteraceae species, *Taraxacum* plastomes (dandelions) [48]. Most of these large repeats were present in the intergenic spacers, but a large proportion was found within the *ycf* gene as in *Ambrosia trifida* (giant ragweed) [54]. Lengths of 20–22 repeats were the most frequent (45%) followed by lengths of 23–26 repeats (37%), with repeats of 27–30 (6%) and > 42 (12%) being quite rare (Figure 5B). 

### 3.3. Sequence Divergence and Hotspots

**Figure 6:**
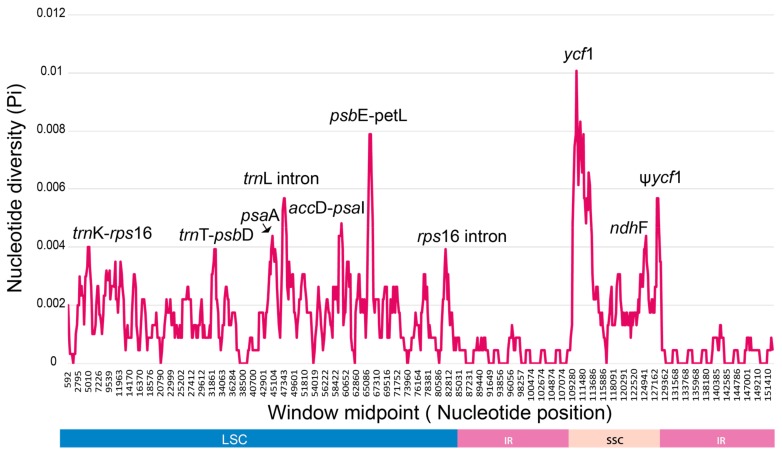
DNA sequence polymorphisms of three *Sonchus* chloroplast genomes (*S. webbii*, *S. asper*, and *S. oleraceus*) calculated using a sliding window analysis of 1000 bases and 200 base step sizes using DNAsp. Ten most divergent regions are suggested as mutation hotspots and potential chloroplast markers for *Sonchus* species.

Nucleotide diversity among five weedy *Sonchus* cp genomes was estimated using DnaSP [38] with a sliding window analysis (window length = 1000 bp and step size = 200 bp excluding sites with alignment gaps). The divergence level was compared to the reference genome of *S. webbii* (Figure 6). Overall nucleotide diversity value (Pi) among *Sonchus* chloroplast genomes including the two closely related species, *S. asper* and *S. oleraceus*, was 0.00117 and ranged from 0 to 0.00807. The SSC region showed the highest nucleotide diversity (0.001629) among the regions of LSC, SSC, and IRs, while the lowest value was in the IR boundary regions (0.000254). Ten divergence hotspots were suggested as the potential chloroplast markers for phylogenetic studies of *Sonchus* species: four intergenic regions (*trn*K-*rps*16, *trn*T-*psb*D, *acc*D-*psa*I, and *psb*E-*pet*L), two intron regions (*trn*L intron and *rps*16 intron), and four protein coding regions (*psa*A, *ycf*1, *ndh*F, and ψ*ycf*1). Most of them are located in the LSC region, but the most variable hotspots with the highest divergence values are in the SSC region. 

**Figure 7:**
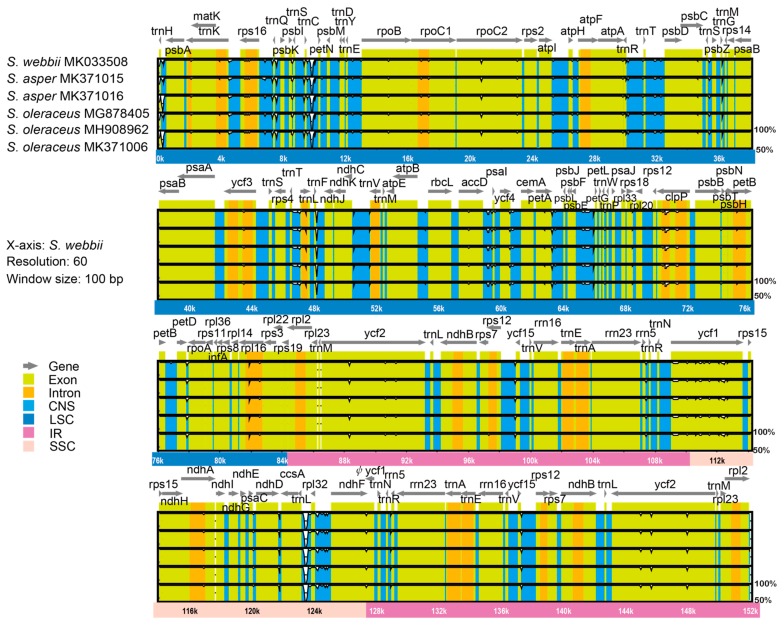
Comparison of five chloroplast genomes of two weedy *Sonchus* species, *S. asper* and *S. oleraceus* plotted against *S. webbii* by mVISTA. Sequence identity is portrayed with a cut-off of 50% identity. The Y-scale axis represents the percent identity within 50%–100%. Grey arrows indicate genes with their orientation and position. Genome regions are color-coded as green blocks for the conserved coding genes (exon), aqua blue blocks for the conserved non-coding sequences in intergenic regions (CNS), and orange blocks for introns. Thick lines below the alignment indicate the quadripartite regions of genomes; the LSC region is in dark blue, IR regions are in pink, and the SSC region is in peach. Black bordered white peaks that are shown in genome regions indicate the divergent regions with sequence variation among *Sonchus* species.

The result of mVISTA plotted against *S. webbii* also exhibited a high degree of synteny and gene order conservation among five weedy *Sonchus* cp genomes. A total of 528 polymorphic sites, which were identified in the DnaSP analysis, were visualized in mVISTA graph from mostly noncoding and intron regions, but also from several protein coding regions. The most divergent coding regions among *Sonchus* cp genomes were *rpo*B, *rpo*C2, *atp*A, *acc*D, *ycf*2, *ndh*F, and *ycf*1 (Figure 7), while the coding genes of *rpo*C1, *rpo*C2, *trn*L, *acc*D, *clp*P, *psb*B, *ndh*D, *ycf*1, *ndh*A, *rps*16, and *ndh*F were presented as the most variable regions between *Taraxacum* and 18 other Asteraceae plastomes [48].

### 3.4. Phylogenetic Analysis

**Figure 8:**
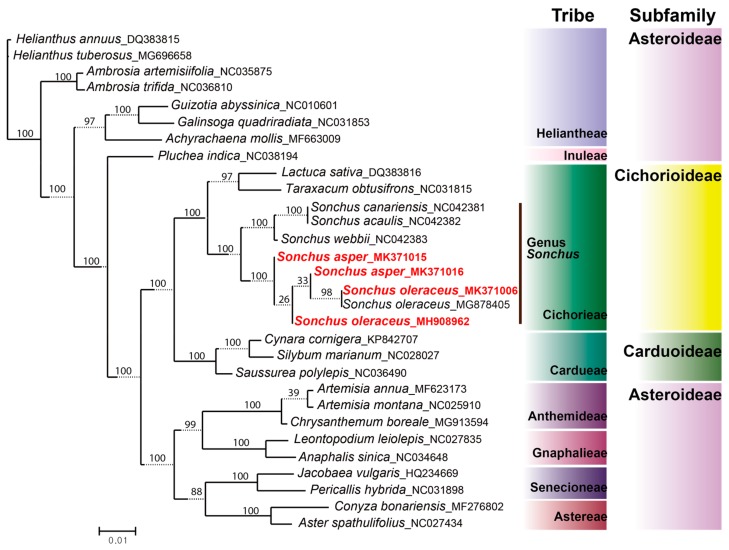
Phylogenetic position and relationships of weedy *Sonchus* sow thistles among Asteraceae species based on whole chloroplast genome sequences inferred from maximum likelihood analysis by IQ-TREE. Numbers above nodes are bootstrap values with 1000 replicates. Newly sequenced four chloroplast genomes representing *S. asper* and *S. oleraceus* in this study are red colored on the phylogenetic tree.

The taxonomic position and evolutionary relationship of *S. asper* and *S. oleraceus* sequenced in this study were assessed by comparative phylogenetic analysis among 30 representative Asteraceae species based on the sequences of whole cp genomes. For overall phylogenetic relationships within the family Asteraceae, the maximum likelihood tree supported the traditional taxonomy of the family, except the subfamily Asteroideae (Figure 8). Asteroideae failed to form a monophyletic clade, as reported in a previous study [55]. Two tribes of Asteroideae, i.e., Heliantheae and Inuleae, were not nested in the monophyletic clade comprising other tribes of the same subfamily, Anthemideae, Gnaphalieae, Senecioneae, and Astereae. With regard to phylogenetic relationships within *Sonchus*, the genus formed a monophyletic clade (100% BS) within the tribe Cichorieae of the subfamily Cichorioideae. *Sonchus* was split into two subclades: i.e., the woody *Sonchus* alliance in the Canary Islands (100% BS) and the weedy *Sonchus* species distributed globally (100% BS). *S. oleraceus* and *S. asper* were nested in the same subclade, being closely related to each other, even though they were not reciprocally monophyletic. The whole chloroplast genome analysis suggested that both *S. asper* and *S. oleraceus* are not monophyletic at the chloroplast level. It is plausible that the species may in fact be monophyletic at the nuclear genome level but is paraphyletic at the chloroplast level, possibly due to incomplete lineage sorting. One lineage of *S. oleraceus* was represented by two accessions which were sampled from geographically widely separated regions (MK371006 from Spain and MG878405 from Australia) (98% BS) and they shared their most recent common ancestor with *S. asper* sampled from Spain (MK371016) (weak 33% BS). On the other hand, the accession of *S. oleraceus* (MH908962) sampled from Dok-do Island in the East Sea (between the Korean peninsula and Japanese archipelago) represented a different plastome type. It was deeply nested within the paraphyletic group of *S. asper* and, interestingly, the overall length of the whole plastome (151,849 bp) was the same as the ones of *S. asper* sampled from Spain (MK371015 and MK371016) (Table 1). We consequently confirmed that the plant material studied from Dok-do Island showed typical morphological characteristics of *S. oleraceus* in its leaves and achenes in order to avoid misinterpretations from our phylogenetic results. The maximum likelihood tree showed that *S. oleraceus* from Dok-do Island (MH908962) is a sister clade containing one accession of *S. asper* (MK371016) and two accessions of *S. oleraceus* (MK371006 from Spain and MG878405 from Australia). Therefore, it is conceivable that *S. oleraceus* accession on Dok-do Island, which contains a distinct plastome type from two other accessions, represents a distinct lineage of *S. oleraceus* that is probably spread throughout Eurasia. Nevertheless, our results do not support the existence of clear geographic patterns for plastome lineages since the two accessions of *S. asper* from Spain (140 km apart, without apparent geographic barriers) seemed to originate from differentiated lineages and the samples of *S. oleraceus* from Spain and Australia (sampled in geographically remote areas) shared the same plastome profile. The current results generally support the hypothesis that *S. asper* contributed as the maternal parent in the hybrid origin of *S. oleraceus* in multiple lineages, a common feature in polyploidy processes [56,57]. However, the origin and evolution of amphidiploid *S. oleraceus* are yet to be determined precisely, which will likely require the inclusion of nuclear DNA analysis and cytological investigation (e.g., fluorescence in situ hybridization, FISH; or genomic in situ hybridization, GISH) to infer the paternal parents.

## 4. Conclusions

In this study, we sequenced, assembled, and annotated four cp genomes of two weedy *Sonchus* sow thistles (*S. asper* and *S. oleraceus*) and analyzed five cp genomes including an additional cp genome of *S. oleraceus* (Australia) obtained from GenBank. The results of comparative genomic analyses revealed that the genomes are highly conserved structurally, sharing most common genomic features of sequences, gene content, numbers, and distributions of repeated sequences despite the morphological and cytological differences between *S. asper* and *S. oleraceus*. The phylogenetic relationship based on whole chloroplast genome sequences suggested that amphidiploid *S. oleraceus* most likely originated multiple times from the closely related congeneric species *S. asper*. We believe that the SSRs, especially longer ones, show potential as useful markers if sample sizes are increased. Lastly, highly variable regions of both coding and noncoding regions were identified as potential phylogenetic markers for *Sonchus* species. 

## Figures and Tables

**Table 1:** Summary of the complete chloroplast genome characteristics of five accessions of two weedy species of *Sonchus*: *S. asper* and *S. oleraceus*.

Characteristics	*S. asper*		*S. oleraceus*		
GenBank Accession Number/Locality ID	MK371015 Spain/VIL-1	MK371016 Spain/MAR-1	MG878405 Australia	MK371006 Spain/MAR-1	MH908962 Korea/Dokdo
Total length (bp)	151,849	151,849	151,808	151,808	151,849
LSC size (bp)	84,156	84,156	84,157	84,113	84,156
SSC size (bp)	24,738	24,738	24,707	24,739	24,738
IR size (bp)	18,217	18,217	18,237	18,217	18,217
Number of genes	130 (17 duplicated in IR)	130 (17 duplicated in IR)	130 (17 duplicated in IR)	130 (17 duplicated in IR)	130 (17 duplicated in IR)
Number of protein coding genes	80 (+7 in IR)	80 (+7 in IR)	80 (+7 in IR)	80 (+7 in IR)	80 (+7 in IR)
Number of tRNA genes	30 (+7 in IR)	30 (+7 in IR)	30 (+7 in IR)	30 (+7 in IR)	30 (+7 in IR)
Number of rRNA genes	3 (+3 in IR)	3 (+3 in IR)	3 (+3 in IR)	3 (+3 in IR)	3 (+3 in IR)
GC content of whole genome (frequency/%)	57,133/37.6%	57,134/37.6%	57,130/37.6%	57,130/37.6%	57,134/37.6%

cpDNA: chloroplast DNA; LSC: large single copy region; SSC: small single copy region; IR: inverted repeat; GC: guanine-cytosine.

**Table 2:** Genes present in the complete chloroplast genomes of five accessions of two weedy species of *Sonchus*: *S. asper* and *S. oleraceus*.

Category	Gene Name
Photosystem I	*psa*A, *psa*B, *psa*C, *psa*I, *psa*J, *ycf*3 ^b^, *ycf*4
Photosystem II	*psb*A, *psb*B, *psb*C, *psb*D, *psb*E, *psb*F, *psb*H, *psb*I, *psb*J, *psb*K, *psb*L, *psb*M, *psb*N, *psb*T, *psb*Z
Cytochrome b6/f complex	*pet*A, *pet*B ^a^, *pet*D, *pet*G, *pet*L, *pet*N
Cytochrome C synthesis	*ccs*A
ATP synthase	*atp*A, *atp*B, *atp*E, *atp*F ^a^, *atp*H, *atp*I
RuBisCO	*rbc*L
NADH oxidoreductase	*Ndh*A ^a^, *ndh*B ^a,c^, *ndh*C, *ndh*D, *ndh*E, *ndh*F, *ndh*G, *ndh*H, *ndh*I, *ndh*J, *ndh*K
Large subunit ribosomal proteins	*rpl*2 ^a,c^, *rpl*14, *rpl*16^a^, *rpl*20, *rpl*22, *rpl*23^c^, *rpl*32, *rpl*33, *rpl*36
Small subunit ribosomal proteins	*rps*2, *rps*3, *rps*4, *rps*7^c^, *rps*8, *rps*11, *rps*12 ^b,^^c,d^, *rps*14, *rps*15, *rps*16 ^a^, *rps*18, *rps*19
RNA polymerase	*rpo*A, *rpo*B, *rpo*C1 ^a^, *rpo*C2
Translation initiation factor	*inf*A
Others	*acc*D, *cem*A, *clp*P ^b^, *mat*K
Unknown function genes (conserved reading frames)	*ycf*1, *ycf* 2 ^c^, *ycf*15 ^c^
Ribosomal RNAs	*rrn*5 ^c^, *rrn*16 ^c^, *rrn*23 ^c^
Transfer RNAs	*trn*A-UGC ^a,c^, *trn*C-GCA, *trn*C-ACA ^a^, *trn*D-GUC, *trn*E-UUC, *trn*E-UUC ^a,c^, *trn*F-GAA, *trn*G-GCC, *trn*G-UCC ^a^, *trn*H-GUG, *trn*K-UUU ^a^, *trn*L-CAA^c^, *trn*L-UAA ^a^, *trn*L-UAG, *trn*M-CAU, *trn*M-CAU, *trn*M-CAU ^c^, *trn*N-GUU ^c^, *trn*P-UGG, *trn*Q-UUG, *trn*R-ACG ^c^, *trn*R-UCU, *trn*S-CGA ^a^, *trn*S-UGA, *trn*S-GGA, *trn*T-GGU, *trn*T-UGU, *trn*V-GAC ^c^, *trn*W-CCA, *trn*Y-GUA

^a^ Gene containing a single intron. ^b^ Gene containing two introns. ^c^ Two gene copies in IRs. ^d^ Trans-splicing gene

## Supplementary Materials

The following are available online at https://www.mdpi.com/2073-4425/10/11/881/s1, Table S1: The frequency of SSR candidates (2), Table S2: SSR candidates.

## References

[1] Holm L.G., Plucknett D.L., Pancho J.V., Herberger J.P. (1977). The World’s Worst Weeds: Distribution and Biology.

[2] Roberts H.A., Neilson J.E. (1981). Seed survival and periodicity of seedling emergence in twelve weedy species of Compositae. Ann. Appl. Biol..

[3] Hassan M.O., Gomaa N.H., Fahmy G.M., González L., Hammouda O., Atteya A.M. (2014). Interactions between *Sonchus oleraceus* L. and some weeds in agroecosystems in Egypt. Ann. Agric. Sci..

[4] Hutchinson I.A., Colosi J., Lewin R.A. (1984). The biology of Canadian weeds. 63. *Sonchus asper* (L.) Hill and *S. oleraceus* L.. Can. J. Plant. Sci..

[5] Boutsalis P., Powles S.B. (1995). Inheritance and mechanism of resistance to herbicides inhibiting acetolactate synthase in *Sonchus oleraceus* L.. Theor. Appl. Genet..

[6] Cook T., Davidson B., Miller R. A new glyphosate resistant weed species confirmed for northern New South Wales and the world: Common sowthistle (*Sonchus oleraceus*). Proceedings of the 19th Australasian Weeds Conference.

[7] Cambie R.C., Ferguson L.R. (2003). Potential functional foods in the traditional Maori diet. Mutat. Res. Fundam. Mol. Mech..

[8] Li X.M., Yang P.L. (2018). Research progress of *Sonchus* species. Int. J. Food Prop..

[9] Kilian N., Gemeinholzer B., Lack H.W., Funk V.A., Susanna A., Stuessy T.F., Bayer R.J. (2009). Cichorieae. Systematics, Evolution, and Biogeography of Compositae.

[10] Kilian N., Hand R., von Raab-Straube E. Cichorieae Systematics Portal. http://cichorieae.e-taxonomy.net/portal/.

[11] Boulos L. (1972). Révision systématique du genre *Sonchus* L. s.l. I. Introduction et classification. Bot. Not..

[12] Mejías J.A., Kim S.-C. (2012). Taxonomic treatment of Cichorieae (Asteraceae) endemic to the Juan Fernandez and Desventuradas Islands (SE Pacific). Ann. Bot. Fenn..

[13] Kim S.-C., Lee C., Mejias J. (2007). A Phylogenetic analysis of chloroplast DNA matK gene and ITS of nrDNA sequences reveals polyphyly of the genus *Sonchus* and new relationships among the subtribe Sonchinae (Asteraceae: Cichorieae). Mol. Phylogenet. Evol..

[14] Boulos L. (1960). Cytotaxonomic studies in the genus *Sonchus* 2. The genus *Sonchus*, a general systematic treatment. Bot. Not..

[15] CABI Invasive Species Compendium, *Sonchus Oleraceus* Datasheet. https://www.cabi.org/isc/datasheet/50584#BE1550BF-EFDC-4477-BBF6-40BF99ECFA6D.

[16] Hsieh T.S., Schooler A.B., Hell A., Nalewaja J.A. (1972). Cytotaxonomic of three *Sonchus* species. Am. J. Bot..

[17] Mejías J.A., Andrés C. (2004). Karyological studies in Iberian *Sonchus* (Asteraceae: Lactuceae): *S. oleraceus*, *S. microcephalus* and *S. asper* and a general discussion. Folia Geobot..

[18] Mulligan G.A. (1957). Chromosome numbers of canadian weeds. I. Can. J. Bot..

[19] Turner B.L., Ellison W.L., King R.M. (1961). Chromosome numbers in the Compositae. IV. North American species with phyletic interpretations. Am. J. Bot..

[20] Walter R., Kuta E. (1971). Cytological and embryological studies in *Sonchus* L.I. *Sonchus asper* (L.) HILL and *Sonchus oleraceus* L.. Acta Biol. Cracoviensia Ser. Bot..

[21] Stebbins G.L., Jenkins J.A., Walters M.S. (1953). Chromosomes and phylogeny in the Compositae, tribe Cichorieae. Univ. Calif. Publ. Bot..

[22] Kim S.-C., Crawford D.J., Jansen R.K. (1996). Phylogenetic relationships among the genera of the subtribe Sonchinae (Asteraceae): Evidence from ITS sequences. Syst. Bot..

[23] Kim S.-C., Crawford D.J., Francisco-Ortega J., Santos-Guerra A. (1996). A common origin for woody *Sonchus* and five related genera in the Macaronesian islands: Molecular evidence for extensive radiation. Proc. Natl. Acad. Sci. USA.

[24] Lee C., Kim S.-C., Lundy K., Santos-Guerra A. (2005). Chloroplast DNA phylogeny of the woody *Sonchus* alliance (Asteraceae: Sonchinae) in the Macaronesian Islands. Am. J. Bot..

[25] Daniell H., Lin C.S., Yu M., Chang W.J. (2016). Chloroplast genomes: Diversity, evolution, and applications in genetic engineering. Genome Biol..

[26] Zhang H., Li C., Miao H., Xiong S. (2013). Insights from the complete chloroplast genome into the evolution of *Sesamum indicum* L.. PLoS ONE.

[27] Zhang Y., Iaffaldano B.J., Zhuang X., Cardina J., Cornish K. (2017). Chloroplast genome resources and molecular markers differentiate rubber dandelion species from weedy relatives. BMC Plant Biol..

[28] Cristescu M.E. (2015). Genetic reconstructions of invasion history. Mol. Ecol..

[29] Gaudeul M., Giraud T., Kiss L., Shykoff J.A. (2011). Nuclear and chloroplast microsatellites show multiple introductions in the worldwide invasion history of common ragweed, *Ambrosia artemissifolia*. PLoS ONE.

[30] Besnard G., Henry P., Wille L., Cooke D., Chapuis E. (2007). On the origin of the invasive olives (*Olea europaea* L., Oleaceae). Heredity.

[31] Parks M., Cronn R., Liston A. (2009). Increasing phylogenetic resolution at low taxonomic levels using massively parallel sequencing of chloroplast genomes. BMC Biol..

[32] Zerbino D.R., Birney E. (2008). Velvet: Algorithms for de novo short read assembly using de Bruijn graphs. Genome Res..

[33] Wyman S.K., Jansen R.K., Boore J.L. (2004). Automatic annotation of organellar genomes with DOGMA. Bioinformatics.

[34] Laslett D., Canback B. (2004). ARAGORN, a program to detect tRNA genes and tmRNA genes in nucleotide sequences. Nucleic Acids Res..

[35] Lagesen K., Hallin P., Rødland E.A., Stærfeldt H.H., Rognes T., Ussery D.W. (2007). RNammer: Consistent annotation of rRNA genes in genomic sequences. Nucleic Acids Res..

[36] Lohse M., Drechsel O., Kahlau S., Bock R. (2013). OrganellarGenomeDRAW—A suite of tools for generating physical maps of plastid and mitochondrial genomes and visualizing expression data sets. Nucleic Acids Res..

[37] Kurtz S., Choudhuri J.V., Ohlebusch E., Schleiermacher C., Stoye J., Giegerich R. (2001). REPuter: The manifold applications of repeat analysis on a genomic scale. Nucleic Acids Res..

[38] Librado P., Rozas J. (2009). DnaSP v5: A software for comprehensive analysis of DNA polymorphism data. Bioinformatics.

[39] Frazer K.A., Pachter L., Poliakov A., Rubin E.M., Dubchak I. (2004). VISTA: Computational tools for comparative genomics. Nucleic Acids Res..

[40] Brudno M., Do C.B., Cooper G.M., Kim M.F., Davydov E., Green E.D., Sidow A., Batzoglou S. (2003). NISC Comparative Sequencing Program. LAGAN and Multi-LAGAN: Efficient Tools for Large-Scale Multiple Alignment of Genomic DNA. Genome Res..

[41] Katoh K., Standley D.M. (2013). MAFFT multiple sequence alignment software version 7: Improvements in performance and usability. Mol. Biol. Evol..

[42] Nguyen L.T., Schmidt H.A., von Haeseler A., Minh B.Q. (2014). IQ-TREE: A fast and effective stochastic algorithm for estimating maximum-likelihood phylogenies. Mol. Biol. Evol..

[43] Kalyaanamoorthy S., Minh B.Q., Wong T.K., von Haeseler A., Jermiin L.S. (2017). ModelFinder: Fast model selection for accurate phylogenetic estimates. Nat. Methods.

[44] Kim K.J., Choi K.S., Jansen R.K. (2005). Two chloroplast DNA inversions originated simultaneously during the early evolution of the sunflower family (Asteraceae). Mol. Biol. Evol..

[45] Timme R.E., Kuehl J.V., Boore J.L., Jansen R.K. (2007). A comparative analysis of the *Lactuca* and *Helianthus* (Asteraceae) plastid genomes: Identification of divergent regions and categorization of shared repeats. Am. J. Bot..

[46] Cho M.S., Yang J.Y., Yang T.J., Kim S.-C. (2019). Evolutionary Comparison of the Chloroplast Genome in the Woody *Sonchus* Alliance (Asteraceae) on the Canary Islands. Genes.

[47] Curci P.L., De Paola D., Danzi D., Vendramin G.G., Sonnante G. (2015). Complete chloroplast genome of the multifunctional crop globe artichoke and comparison with other Asteraceae. PLoS ONE.

[48] Salih R.H., Majeský Ľ., Schwarzacher T., Gornall R., Heslop-Harrison P. (2017). Complete chloroplast genomes from apomictic *Taraxacum* (Asteraceae): Identity and variation between three microspecies. PLoS ONE.

[49] Wang M., Cui L., Feng K., Deng P., Du X., Wan F., Weining S., Nie X. (2015). Comparative analysis of Asteraceae chloroplast genomes: Structural organization, RNA editing and evolution. Plant. Mol. Biol. Rep..

[50] Ogihara Y., Terachi T., Sasakuma T. (1988). Intramolecular recombination of chloroplast genome mediated by short direct-repeat sequences in wheat species. Proc. Natl. Acad. Sci. USA.

[51] Milligan B.G., Hampton J.N., Palmer J.D. (1989). Dispersed repeats and structural reorganization in subclover chloroplast DNA. Mol. Biol. Evol..

[52] Wang M.L., Barkley N.A., Jenkins T.M. (2009). Microsatellite markers in plants and insects. Part I: Applications of biotechnology. Genesgenomes Genom..

[53] Thiel T., Michalek W., Varshney R., Graner A. (2003). Exploiting EST databases for the development and characterization of gene-derived SSR-markers in barley (*Hordeum vulgare* L.). Theor. Appl. Genet..

[54] Sablok G., Amiryousefi A., He X., Hyvönen J., Poczai P. (2019). Sequencing the plastid genome of giant ragweed (*Ambrosia trifida*, Asteraceae) from a herbarium specimen. Front. Plant. Sci..

[55] Wang X.Y., Zhou Z.S., Liu G., Qian Z.Q. (2018). Characterization of the complete chloroplast genome of the invasive weed *Galinsoga quadriradiata* (Asterales: Asteraceae). Conserv. Genet. Resour..

[56] Soltis D.E., Soltis P.S. (1999). Polyploidy: Recurrent formation and genome evolution. Trends Ecol. Evol..

[57] Soltis D.E., Soltis P.S., Pires J.C., Kovaric A., Tate J.A., Mavrodiev E. (2004). Recent and recurrent polyploidy in *Tragopogon* (Asteraceae): Cytogenetic, genomic and genetic comparisons. Biol. J. Linn. Soc..

